# ^1^H, ^13^C, and ^15^N backbone chemical shift assignments of coronavirus-2 non-structural protein Nsp10

**DOI:** 10.1007/s12104-020-09984-1

**Published:** 2020-11-07

**Authors:** N. Kubatova, N. S. Qureshi, N. Altincekic, R. Abele, J. K. Bains, B. Ceylan, J. Ferner, C. Fuks, B. Hargittay, M. T. Hutchison, V. de Jesus, F. Kutz, M. A. Wirtz Martin, N. Meiser, V. Linhard, D. J. Pyper, S. Trucks, B. Fürtig, M. Hengesbach, F. Löhr, C. Richter, K. Saxena, A. Schlundt, H. Schwalbe, S. Sreeramulu, A. Wacker, J. E. Weigand, J. Wirmer-Bartoschek, J. Wöhnert

**Affiliations:** 1 Institute for Organic Chemistry and Chemical Biology, Johann Wolfgang Goethe-University Frankfurt, Max-von-Laue-Str. 7, 60438 Frankfurt/M, Germany; 2 Institute for Biochemistry, Biocentre, Johann Wolfgang Goethe-University Frankfurt, Max-von-Laue-Str. 7, 60438 Frankfurt/M, Germany; 3 Institute for Molecular Biosciences, Johann Wolfgang Goethe-University, Max-von-Laue-Str. 7, 60438 Frankfurt/M, Germany; 4 Institute of Biophysical Chemistry, Center for Biomolecular Magnetic Resonance (BMRZ), Johann Wolfgang Goethe-University, Max-von-Laue-Str. 7, 60438 Frankfurt/M, Germany; 5 Department of Biology, Technical University of Darmstadt, Schnittspahnstr 10, 64287 Darmstadt, Germany

**Keywords:** SARS-CoV-2, Non-structural protein, Solution NMR-spectroscopy, Covid19-NMR

## Abstract

The international Covid19-NMR consortium aims at the comprehensive spectroscopic characterization of SARS-CoV-2 RNA elements and proteins and will provide NMR chemical shift assignments of the molecular components of this virus. The SARS-CoV-2 genome encodes approximately 30 different proteins. Four of these proteins are involved in forming the viral envelope or in the packaging of the RNA genome and are therefore called structural proteins. The other proteins fulfill a variety of functions during the viral life cycle and comprise the so-called non-structural proteins (nsps). Here, we report the near-complete NMR resonance assignment for the backbone chemical shifts of the non-structural protein 10 (nsp10). Nsp10 is part of the viral replication-transcription complex (RTC). It aids in synthesizing and modifying the genomic and subgenomic RNAs. Via its interaction with nsp14, it ensures transcriptional fidelity of the RNA-dependent RNA polymerase, and through its stimulation of the methyltransferase activity of nsp16, it aids in synthesizing the RNA cap structures which protect the viral RNAs from being recognized by the innate immune system. Both of these functions can be potentially targeted by drugs. Our data will aid in performing additional NMR-based characterizations, and provide a basis for the identification of possible small molecule ligands interfering with nsp10 exerting its essential role in viral replication.

## Biological context

The current worldwide COVID-19 pandemic caused by the severe acute respiratory syndrome coronavirus 2 (SARS-CoV-2) has severely impacted nearly every area of human life. In the absence of reliable therapeutic options or vaccination, research efforts have been increased in order to understand molecular characteristics of the functional components of the virus. SARS-CoV-2 belongs to the family of *Coronaviridae*, whose members share a distinct pattern in their genomic organization (Lai 1990; Snijder et al. 2016). In contrast to several other viruses, the large (+)-strand RNA genome of ~ 30 kb requires high fidelity genome replication while offering coding space for additional proteins.

The RNA genome of SARS-CoV-2 encodes at least 14 polypeptides, some of which are cleaved by viral proteases into their functional forms. The non-structural protein 10 (nsp10) is generated from the first open reading frame of the virus (ORF1) through cleavage by the main protease nsp5 to yield a 139 amino acid protein of 14.8 kDa.

Previous work has been reported on the homologous proteins of nsp10 from SARS-CoV (Bouvet et al. 2014), middle eastern respiratory syndrome coronavirus (MERS-CoV) (Aouadi et al. 2017) and murine hepatitis virus (MHV) (Matthes et al. 2006).

Nsp10 plays numerous roles in coronavirus replication. As part of the replicase complex, it interacts with nsp14 (Minskaia et al. 2006) to stimulate the exonuclease activity of nsp14 (Ferron et al. 2017) and thus contributes to the enhanced replication fidelity of coronaviruses in comparison to other RNA viruses. It also increases the catalytic activity of nsp16 (Bouvet et al. 2010), which methylates the 2′-hydroxyl group of the +1 adenosine of genomic and subgenomic (+)-strand RNAs. The presence of this modification prevents the recognition and degradation of viral RNAs by the innate immune system of the host (Daffis et al. 2010; Züst et al. 2011). Additional regulatory functions for nsp10, such as interactions with nsp1 or nsp7, have been proposed (Brockway et al. 2004), but these functionalities require additional characterization. In summary, the functional diversity of nsp10 during the viral life cycle renders it a promising drug target (Wang et al. 2015).

Several structures of nsp10 from SARS-CoV have previously been solved both in isolation (Joseph et al. 2006; Su et al. 2006) and in complex with either nsp14 (Bouvet et al. 2012; Ma et al. 2015) or nsp16 (Chen et al. 2011; Decroly et al. 2011). The structures showed the presence of a completely novel fold including two unusual zinc finger motifs. The first zinc finger includes residues C74, C77, H81 and C90 (SARS-CoV numbering) and is loosely characterized as a Zn^2+^-binding loop. The second zinc-binding motif involving C117, C120, C128, and C130 can be classified as a “gag-knuckle” type motif (Krishna et al. 2003). All of these residues can also be found in the nsp10 sequence of SARS-CoV-2 (Krafcikova et al. 2020; Viswanathan et al. 2020). Here, we provide a near complete assignment of nsp10 backbone NMR resonances. Due to the known interaction sites with other proteins, this assignment will aid further NMR-based structural investigations as well as ligand binding studies.

## Methods and experiments

### Construct design

The amino acid sequence of SARS-CoV-2 nsp10 was obtained from the NCBI reference genome entry NC_045512.2, identical to GenBank entry MN908947.3 (Wu et al. 2020). The gene was codon-optimized for expression in *E. coli*, commercially synthesized and sub-cloned into a pET21b(+) vector (*Genscript*), carrying an additional sequence at the N-terminus (MGSDKIHHHHHH) including a hexa-histidine (His_6_)-tag for purification.

### Sample preparation

The pET21b(+) plasmid containing the nsp10 sequence was transformed into T7-Express *E. coli* cells. Nsp10 was heterologously expressed with an N-terminal His_6_-tag (Joseph et al. 2006). Uniformly ^13^C,^15^N-labeled nsp10 protein was expressed in M9 minimal medium containing 1 g/L ^15^NH_4_Cl, 2 g/L ^13^C_6_-d-glucose and 100 mg/L ampicillin. After the OD_600_ reached a value between 0.6 and 0.7, the culture was induced with 0.5 mM isopropyl-β-d-thiogalactoside (IPTG) and supplemented with 50 µM ZnCl_2_. The final preparative expression was performed at 20 °C, 120 rpm for 12 h. Cells from a 2 L culture were harvested at 4000 g for 15 min using a Beckmann centrifuge with a JLA 8.1000 rotor. The pellet was resuspended in 100 mL of buffer A (25 mM Tris pH 8, 300 mM NaCl, 5 mM imidazole, 10 mM β-mercaptoethanol) and supplemented with 50 µM ZnCl_2_, and two protease-inhibitor tablets (*cOmplete™, Roche, Germany*). Cells were mechanically lysed using Microfluidics M-110P at 15,000 PSI (pounds per square inch) under continuous ice cooling, followed by centrifugation at 4 °C and 38,400×*g* for 45 min using a Beckmann centrifuge with a JA 20 rotor.

The supernatant was further purified using immobilized metal ion affinity chromatography (IMAC) followed by size exclusion chromatography (SEC). After centrifugation, the supernatant was loaded onto a 5 mL HisTrap HP column (*GE Healthcare, USA*) connected to an FPLC system (*Äktapurifier™, GE Healthcare, USA*). Elution of the bound protein was achieved with a linear gradient of 500 mM imidazole containing buffer A. Protein-containing fractions were combined, concentrated using centrifugal concentrator devices (*VivaSpin20*, *Sartorius, Germany, MWCO 10000*) and further purified with a 320 mL HiLoad 26/600 Superdex 75 pg gel filtration column (*GE Healthcare, USA*) using 50 mM sodium phosphate buffer (pH 7.5), 50 mM NaCl, 5 mM dithiothreitol (DTT). Purity of the produced protein was verified by SDS-PAGE analysis and confirmed using mass spectrometry (MALDI). Finally, the protein was concentrated using centrifugal concentrator devices (*VivaSpin20*, *Sartorius, Germany, MWCO 10000*).

SEC-MALS analysis was performed using a Superdex 75, 10/300 GL column at a flow rate of 0.5 mL/min, using a Wyatt miniDAWN TREOS with a 658 nm laser. Refractive index was monitored using a Wyatt Optilab rEX.

### NMR experiments

The protein samples were measured in NMR buffer containing 50 mM phosphate buffer pH 7.5, 50 mM NaCl, 5 mM DTT, 95% H_2_O/5% D_2_O. Spectra were recorded at 298 K on Bruker spectrometers ranging from 600 to 950 MHz equipped with z-axis gradient ^1^H{^13^C,^15^N} triple resonance cryogenic probes and on a Bruker 500 MHz spectrometer equipped with a room-temperature triple-resonance probe. The spectrometer was locked on D_2_O. ^1^H chemical shifts were referenced to DSS at 0.00 ppm and ^13^C,^15^N chemical shifts were calculated relative to the ^1^H frequency according to (Wishart 2011). All NMR spectra were processed by using TopSpin version 3.2 (Bruker Biospin) and analyzed and visualized with SPARKY version 3.114 (Lee et al. 2015). Parameters of the NMR experiments and spectrometers used in this study are listed in Table 1.

**Table 1:** List of experiments collected to perform the sequence specific assignment of nsp10. Main parameters used are reported

Experiments	Time domain data size (points)			Spectral width (ppm)			Number of scans	Delay time (s)	NUS %
	T_1_	T_2_	T_3_	F_1_	F_2_	F_3_			
^1^H,^15^N BEST-TROSY	1024	1272		50 (^15^N)	10.0 (^1^H)		4	0.3	
BEST-TROSY-HN(CO)CACB	208	256	1272	59.7 (^13^C)	26.0 (^15^N)	10.0 (^1^H)	8	0.3	
BEST-TROSY-HNCACB	216	256	1272	59.7 (^13^C)	26.0 (^15^N)	10.0 (^1^H)	8	0.3	
BEST-TROSY-HN(CA)CO	216	256	1272	12.0 (^13^C)	26.0 (^15^N)	10.0 (^1^H)	8	0.3	25
BEST-TROSY-HNCO	216	256	1272	12.0 (^13^C)	26.0 (^15^N)	10.0 (^1^H)	4	0.3	
^15^N R_1_	12	256	2048	–	28.0 (^15^N)	16.0 (^1^H)	8	2.0	
^15^N R_2_	12	256	2048	–	28.0 (^15^N)	16.0 (^1^H)	8	2.0	
^15^N-NOE	2	256	2048	–	28.0 (^15^N)	16.0 (^1^H)	80	10	
TRACT	28	96	896	–	30.0 (^15^N)	12.0 (^1^H)	8–40	0.3	

For the sequential backbone resonances assignment, a set of 3D NMR experiments including BEST-TROSY (Farjon et al. 2009; Solyom et al. 2013) based HNCACB, HN(CO)CACB, HNCO, HN(CA)CO experiments were used.

For the temperature series, 2D ^1^H,^15^N BEST-TROSY spectra were measured from 293 to 308 K with 5 K increments on a 600 MHz spectrometer. Temperature coefficients of the amide protons were calculated from a linear fit of a chemical shift perturbation as a function of a temperature (Baxter and Williamson 1997).

Heteronuclear ^15^N relaxation experiments ({^1^H}-^15^N hetNOE, T_1_ and T_2_) were performed at 600 MHz and 298 K using a 0.15 mM and a 1.6 mM sample. For determining the longitudinal T_1_^15^N relaxation time, a series of spectra with the following relaxation delays was used: 20, 60, 100, 200, 400, 600, 800, 1200, 1400, 1600, 1800 ms. The transverse ^15^N relaxation time T_2_ was determined from spectra with the following relaxation delays: 16.96, 33.92, 67.84, 101.76, 135.68, 169.60, 203.52, 271.36 ms. The {^1^H}-^15^N hetNOEs were calculated from the signal intensity ratio (I_on_/I_off_) obtained from spectra recorded with and without saturation of amide protons with a recovery (d_1_) and saturation delay of 10 s.

TRACT experiments (Lee et al. 2006) were carried out at 500 MHz to determine rotational correlation times at four different protein concentrations (1.2 mM, 0.6 mM, 0.3 mM and 0.15 mM). A two-dimensional BEST version (Rennella and Brutscher 2013) was employed to avoid an underestimation of the correlation time by the contribution of intense signals from mobile residues. A total of 21 well-resolved cross peaks from structured regions of the protein were chosen for evaluation.

### Assignments and data deposition

The backbone chemical shift assignment of nsp10 protein was conducted manually by using standard double- and triple-resonance NMR experiments on uniformly labeled samples at 298 K. With the heteronuclear 3D experiments listed above we could assign 89% of the ^1^H,^15^N amide signals and 94% of the total backbone assignment (C_α_—93%, C_β_—95%, C′—92%). When excluding the first twelve N-terminal residues including the His_6_-tag, the backbone assignment comprises 92% of the ^1^H,^15^N pairs and 95.5% of the total resonances (C_α_—96%, C_β_—95%, C′—96%). Residues M1, G2, F28, C29, T59 H60 and the N-terminal His_6_-tag were not assigned. Assignment of the residues K5 and W135 is tentative.

**Fig. 1:**
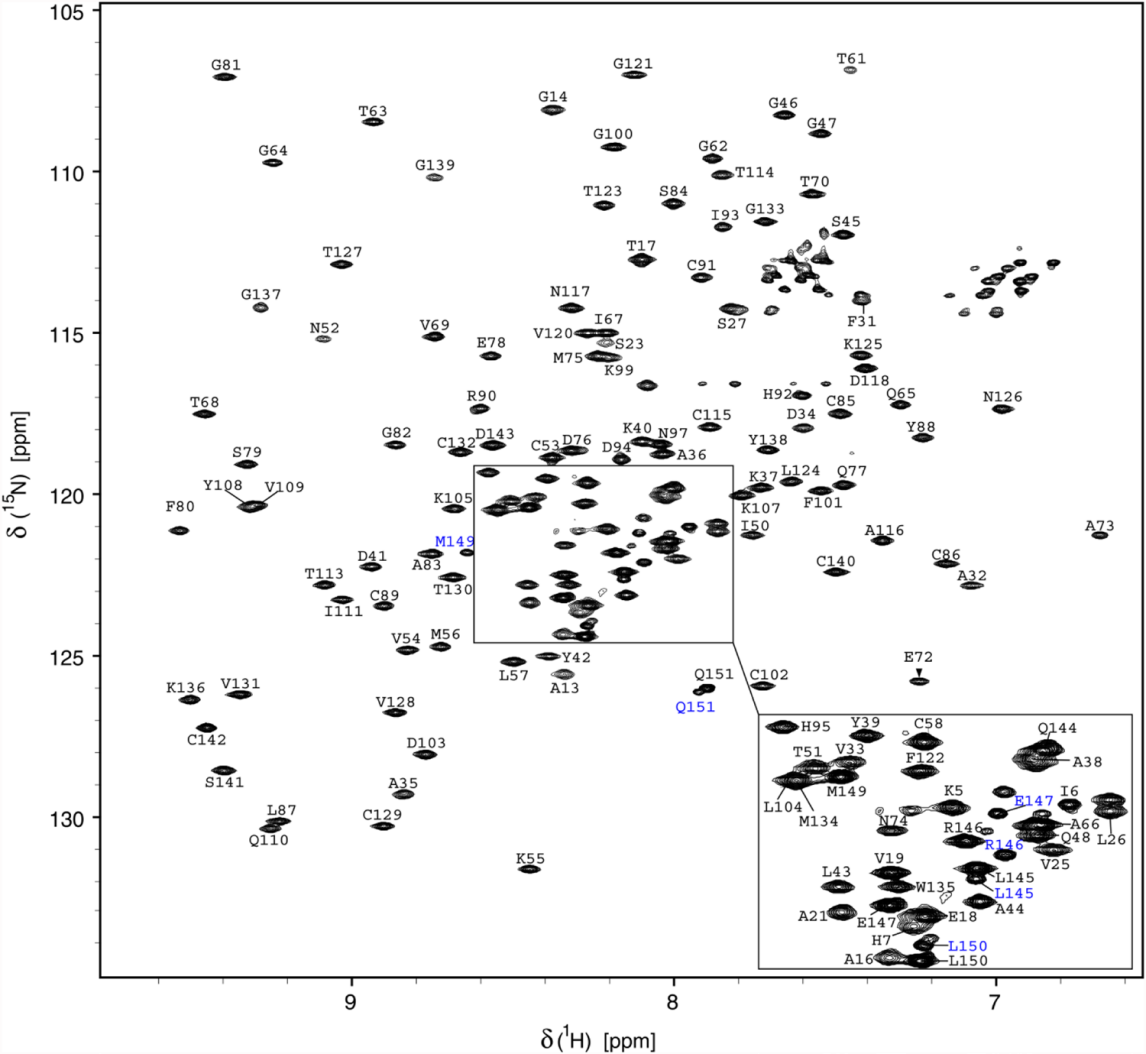
^1^H,^15^N-BEST-TROSY spectrum of ^13^C,^15^N-labeled SARS-CoV-2 nsp10 (0.4 mM) in 50 mM phosphate buffer pH 7.5, 50 mM NaCl, 5 mM DTT, and 5% D_2_O measured at 298 K on a 950 MHz spectrometer. Backbone resonance assignment labels are given. The minor conformation adopted by the nine C-terminal residues is highlighted in blue

The number of signals observed in BEST-TROSY experiments is approximately 5% higher than expected for the nsp10 protein. Lower intensities for a subset of the signals are indicative of the presence of a second minor conformation in slow exchange on the NMR time scale. The resonances in question were assigned to the last seven C-terminal amino acids (Fig. 1). The backbone resonance assignment for the main conformation was deposited in the biological magnetic resonance bank (BMRB ID: 50392).

### Study of nsp10 dynamics

**Fig. 2:**
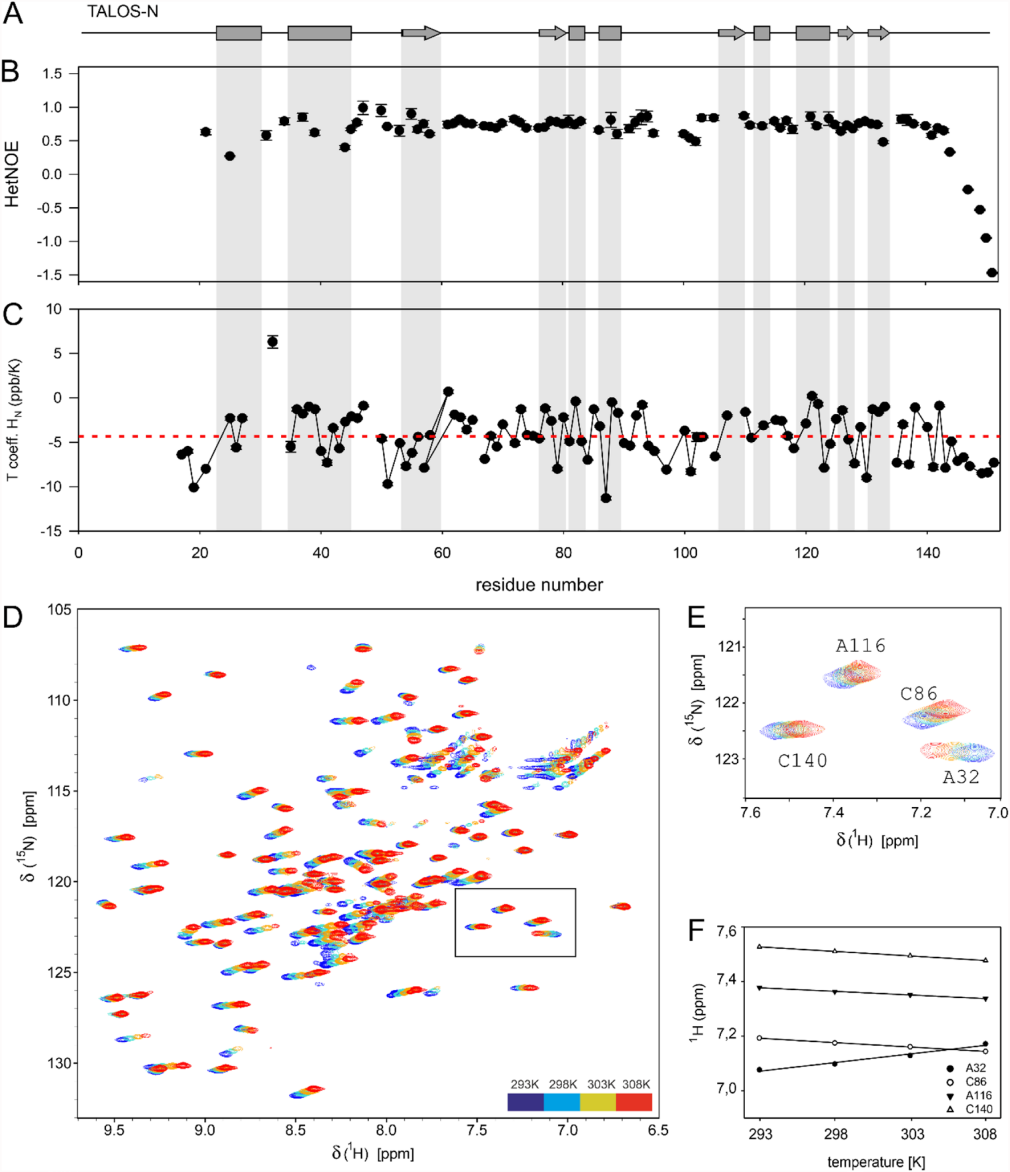
Backbone dynamics of nsp10. **a** Schematic representation of an NMR chemical-shift-based TALOS-N secondary-structure prediction of nsp10. **b** {1H}-^15^N heteronuclear NOEs measured at 298 K and 0.15 mM nsp10 are shown as a function of residue number. **c** The temperature coefficients (*T*_coeff_) determined from a series of 2D ^1^H,^15^N BEST-TROSY spectra measured from 293 to 308 K with 5 K increments at 600 MHz. **d** Overlay of 2D ^1^H,^15^N BEST-TROSY spectra recorded at 600 MHz at different temperatures (color code is shown in the figure). **e** Selected zoom-in showing residues A32, C86, A116 and C140, representing different hydrogen bond strengths. **f** Plot of the amide proton chemical shift (in ppm) as a function of temperature (in K)

We also characterized the dynamics of nsp10 by performing {^1^H}^15^N heteronuclear NOE experiments for two sample concentrations (1.6 mM and 0.15 mM) and analyzing intramolecular hydrogen bonds using temperature factors (Fig. 2). The last nine C-terminal residues from the major conformation show low heteronuclear NOEs values, indicating the high flexibility for this region (Fig. 2b).

**Fig. 3:**
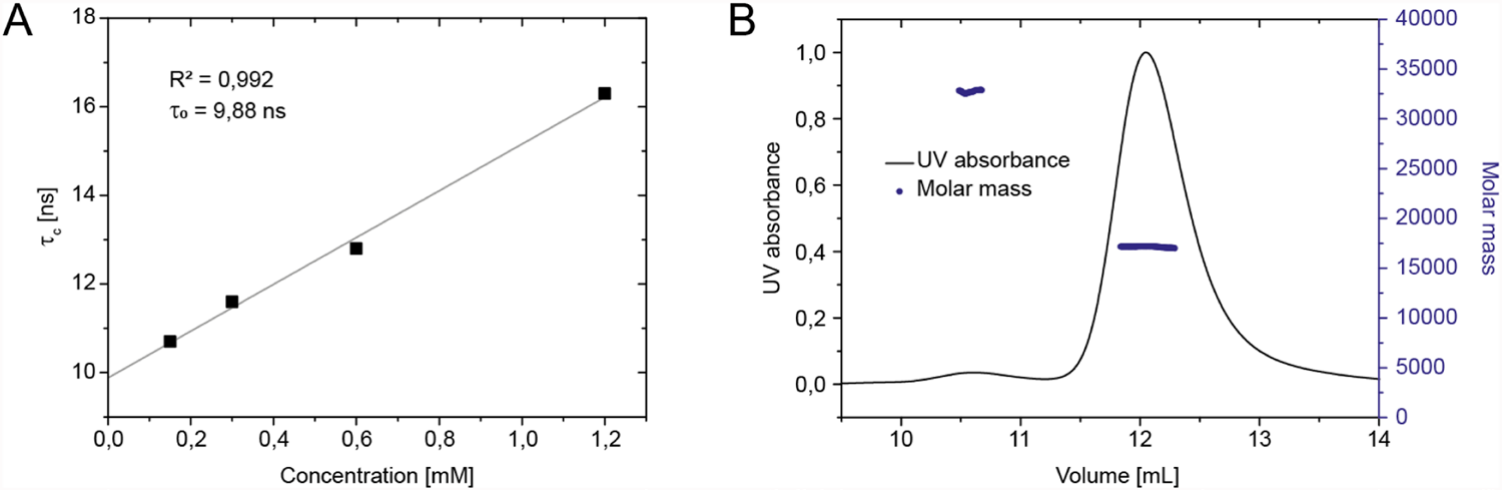
Oligomerization analysis. **a** TRACT analysis. Linear fit of τ_c_ against protein concentration from data points at 0.15, 0.3, 0.6, and 1.2 mM nsp10. **b** SEC-MALS analysis performed at 1.2 mM nsp10 shows a minor amount of oligomerization, which can be assigned to the nsp10 dimer by mass determination

Intramolecular hydrogen bonding was analyzed by monitoring the amide protons chemical shift changes as a function of temperature. Thus, a series of 2D ^1^H,^15^N BEST-TROSY spectra were recorded for a temperature range from 293 to 308 K in 5 K increments. Temperature coefficients calculated from a linear fit of the chemical shift changes with values below—4.5 ppb/K are indicative for fast exchangeable, not hydrogen-bonded amide protons, and temperature coefficients higher than—4.5 ppb/K are characteristic for the involvement of this amide resonance in hydrogen bonding (Baxter and Williamson 1997). The temperature coefficients of the C-terminus of nsp10 are consistent with the relaxation data, supporting a dynamic nature of this part of the protein. The amide group of residue A32 located in the N-terminal region shows an extremely high temperature coefficient with a positive value of 6.3 ± 0.7 ppb/K, the reason of which is not clear (Fig. 2e, f). Furthermore, we performed TRACT measurements (Lee et al. 2006) at different concentrations and determined concentration-dependent oligomerization (Fig. 3a). Experimental τ_c_ for the low concentration sample (0.15 mM) is 10.7 ns, which is agreement with the monomer value predicted by HydroNMR (10.8 ns) (de la Torre et al. 2000). Analysis of the oligomerization status by size exclusion chromatography coupled to multiple angle light scattering (SEC-MALS) (Fig. 3b) shows that a minor fraction of proteins forms higher order structures, whose mass corresponds to a protein dimer.

### Structural comparison of nsp10 from SARS-CoV and SARS-CoV-2

**Fig. 4:**
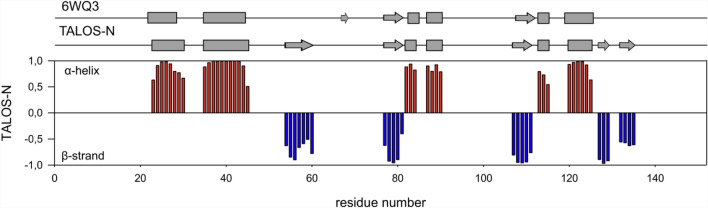
Schematic representation of an NMR chemical-shift-based TALOS-N secondary-structure prediction of nsp10 and its comparison with the secondary structure elements obtained from the X-ray structure extracted from PDB entry 6WQ3, which shows 100% sequence identity between residues S23 and Q144

The backbone resonance assignment was used to predict secondary structure elements of nsp10 using TALOS-N (Shen and Bax 2013). Six α-helixes and five β-strands were identified. Experimentally determined structural elements were compared with motifs obtained from the X-ray structure of SARS-CoV nsp10 (PDB: 6WQ3) (Fig. 4).

**Fig. 5:**
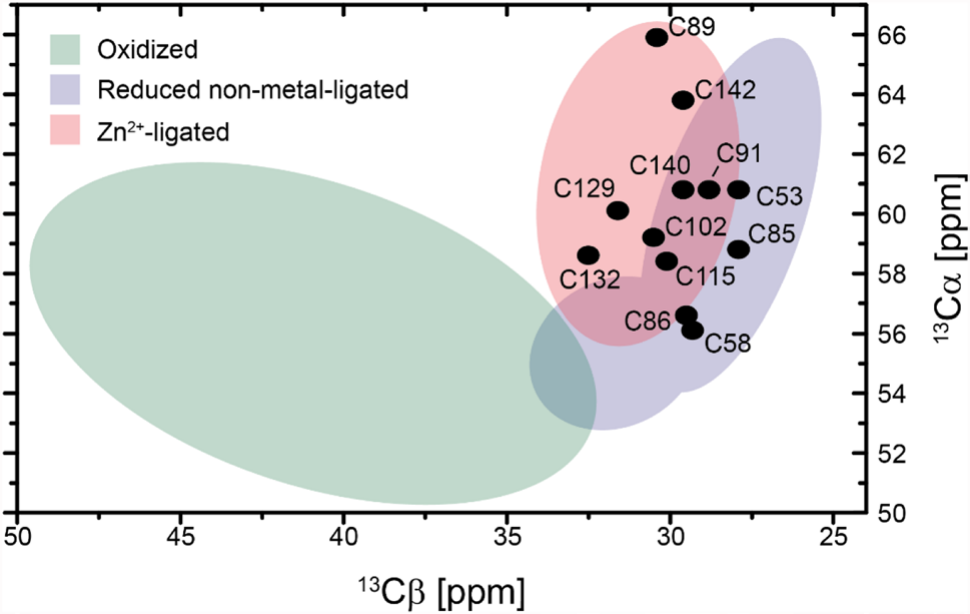
^13^Cα and ^13^Cβ chemical shifts of the assigned nsp10 cysteines. The regions for different chemical states of the cysteines (colored areas) are derived from (Kornhaber et al. 2006)

Overall, the structural elements of both proteins are similar. Differences can be detected in the location of a stretch of amino acids that adopts a β-strand conformation according to the TALOS-N analysis (aa 54–60), which is directly involved in interactions with the complex partner nsp16 in the crystal structure (PDB 6WQ3). A second difference can be observed for some of the C-terminal amino acids, which adopt a zinc finger fold, but are classified as ß-strands in the TALOS prediction. However, all of the assigned cysteine residues show Cβ chemical shifts that are indicative of a Zn^2+^-ligated form (Fig. 5) (Kornhaber et al. 2006).
